# Tungiasis: Participation of Cats and Chickens in the Dispersion and Maintenance of the Disease in an Endemic Tourist Area in Brazil

**DOI:** 10.3390/tropicalmed8100456

**Published:** 2023-09-25

**Authors:** Jamille Bispo de Carvalho Teixeira, Katharine Costa dos Santos, Paula Elisa Brandão Guedes, Rebeca Costa Vitor, Thammy Vieira Bitar, Tatiani Vitor Harvey, Anaiá da Paixão Sevá, Renata Santiago Alberto Carlos

**Affiliations:** 1 Programa de Pós-Graduação em Ciência Animal—PPGCA, Departamento de Ciências Agrárias e Ambientais (DCAA), Universidade Estadual de Santa Cruz (UESC), Ilhéus 45662-900, Bahia, Brazil; jbcarvalho@uesc.br (J.B.d.C.T.); kathycosta95@gmail.com (K.C.d.S.); paulaebg@gmail.com (P.E.B.G.); rebeca.scosta@hotmail.com (R.C.V.); apseva@uesc.br (A.d.P.S.); 2 Departamento de Ciências Agrárias e Ambientais (DCAA), Curso de Medicina Veterinária, Universidade Estadual de Santa Cruz (UESC), Ilhéus 45662-900, Bahia, Brazil; thammy.bittar@gmail.com; 3 Veterinarian, College Station, TX 77845, USA; tvharveyvet@gmail.com

**Keywords:** sand flea, ectoparasite, *Felis catus*, Gallus gallus domesticus, zoonosis

## Abstract

*Tunga* spp. are fleas commonly found in impoverished tropical regions. In Vila Juerana, a tourist community in Ilheus, Bahia, Brazil, where tungiasis is endemic, dogs are the main host of fleas during their life cycle. However, there is no information about the role of cats and chickens in tungiasis in the village. Of the 272 households investigated, 112 had domestic animals, 48 had only dogs, 28 had only cats, and nine had only chickens. Of the 27 households with cohabitation among species, 16 had cats and dogs, eight had chickens and dogs, and three had dogs, cats, and chickens. The injuries due to tungiasis were ranked according to the Fortaleza classification, considering stages I, II, and III as viable lesions. The paws/feet of 71/111 (63.9%) cats and 173/439 (39.4%) chickens were inspected. Dogs that lived with positive cats and chickens also were inspected. Among the 38% (27/7; 95% IC 26.74–49.32) positive cats, 16 cohabited houses with infected dogs but none lived with positive chickens. Of the chickens, 2.3% (4/173; 95% IC 0.07–4.5) had lesions caused by tungiasis. In each household where a cat was infected, the dog was also positive. Two chickens cohabited with an infected dog and the other two did not coexist with other species. Cohabitation with infected dogs and the absence of house confinement restrictions in Vila Juerana make cats important carriers that spread tungiasis in this community. Chickens had a low frequency of tungiasis lesions despite living in proximity to infected dogs and cats.

## 1. Introduction

Tungiasis is a zoonotic disease that mainly affects socially vulnerable populations in slums, rural and coastal settlements, and fishing and indigenous villages [1,2,3,4,5,6]. Despite the low mortality rate of the disease, the morbidity rate is high, due largely to the negligence of public health authorities in addressing the infection [7,8,9,10,11,12,13,14].

In Brazil, the main species associated with the disease in humans and animals is *T. penetrans* [15,16]. The gravid female flea initially penetrates the epidermis (usually of the feet or paws) of its host, seeks a blood source in the tissue for its food, and, after fertilization, undergoes body hypertrophy, increasing five to ten-fold in size and reaching up to 1 cm in diameter. The enlarged fleas are called neosomes [10,11,12,13,14]. Subsequently, the fleas begin oviposition for a period of approximately 21 days, after this period they die [15,16]. The lesions that occur after the flea penetrates a host can cause clinical complications such as pain, swelling, and itching, which can progress to tissue necrosis, secondary bacterial infection, sepsis, loss of digits, or tetanus [3,17,18,19]. Infection in humans is associated with the contact of *T. penetrans* dispersed in the soil with the skin, especially in people living with infected domestic animals such as dogs, pigs, goats, cats, and chickens [10,20,21].

To rank the stage of flea penetration lesions, the Fortaleza classification is used [22], which was created based on observation of the disease in humans and later applied in studies of tungiasis in dogs [6,23].

In sub-Saharan Africa, pigs, dogs, and goats are of great importance in the epidemiological chain of the disease, with pigs being identified as the main hosts and reservoirs of the epidemiological cycle of *Tunga* spp. [24,25,26,27,28,29]. Among Brazilian studies, dogs were identified as the main hosts of *Tunga* spp. in various regions of the country [6,30,31]. Studies carried out in the northeast region also observed the presence of *Tunga* spp. in cats [32,33] and rodents [32]. Cattle [34] and wild animals [35,36] were also reported as suffering from tungiasis in South America.

Depending on the region studied, other animal species may act as reservoirs for *Tunga* spp., as previously reported. For this reason, it is necessary to elucidate the composition of the epidemiological chain in endemic areas in order to contribute to improvements in mitigation strategies for preventing and controlling human infection. In this type of environment, the infection of different animal species can be one of the main obstacles to controlling human and domestic animal infections.

This article analyzes the co-participation of cats and chickens cohabiting with each other and with other animals as maintainers and dispersers of tungiasis in an endemic tourist community in the municipality of Ilheus, Bahia, Brazil.

## 2. Materials and Methods

### 2.1. Ethical Considerations and Study Area

This study was carried out after approval by the Ethics Committee on Animal Use (CEUA) of the State University of Santa Cruz (UESC) under numbers 015/21 and 027/21, and after written consent from the owners of the animals included in this study.

**Figure 1:**
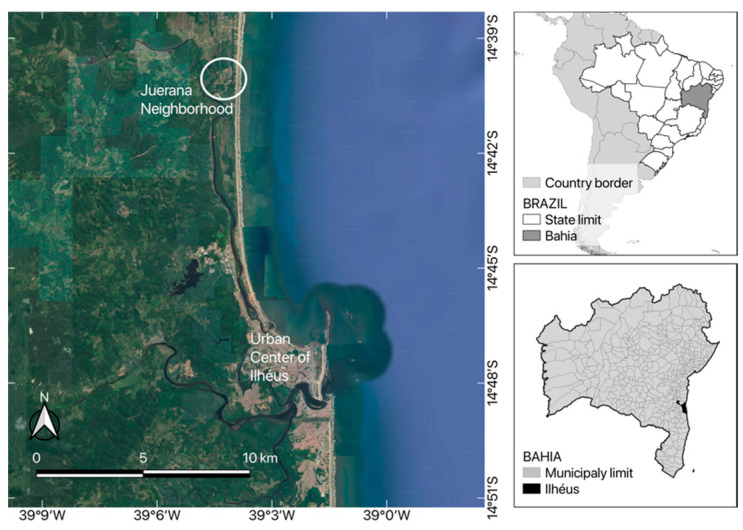
Geolocation of Vila Juerana, Municipality of Ilheus, Bahia, Brazil.

The project was developed in a semi-rural endemic coastal community frequented by tourists (Figure 1), Vila Juerana, located in the district of Aritagua, part of the municipality of Ilheus, Bahia, Brazil (S 14.96767°, W 039.32436°) [37]. The predominant vegetation type is Atlantic Forest, with the presence of beaches and mangrove forests. The climate is classified as humid tropical. This area was previously studied [6,23,37,38] and found to be an endemic zone for tungiasis, with a prevalence of 62.3% (95% CI: 52.7–71.2%) *Tunga* spp. in dogs, causing large numbers of lesions [23].

### 2.2. Clinical Study

A census was conducted that included all residences in Vila Juerana, which were visited to check for the presence of cats, chickens, and any other domestic or wild animals cohabiting with each other and with dogs. During a visit, the owner of the residence was questioned about the presence and description of animal species in their residence. After this stage, houses that had cats, chickens, ducks, tortoises, and parakeets were inspected, and if there were also dogs in these houses, they were also inspected. Each animal (cat or chicken) was considered as a sample unit. Cats, chickens, dogs, ducks, tortoises, and parakeets were physically restrained and inspected to identify lesions caused by *Tunga penetrans*. Animals that did not allow restraint were not inspected. Each visited dwelling included in this study was georeferenced using a Garmin^®^ GPS map device with the UTM system.

Chickens for examination were selected by the owners, who used docility as a criterion, which facilitated manual restraint. Many chickens also had free access to yards and streets, making their capture impossible.

Lesions were counted and staged according to the Fortaleza classification [22] as follows: (a) stage I: penetration of *Tunga* spp., indicated by a red-brown spot approximately 1 mm in size; (b) stage II: full penetration, denoted by a central brownish or black dot and an expanding mother-of-pearl-like halo of 0.5–2.0 mm with indistinct edges and a zone of perilesional erythema; (c) stage III: hypertrophy of the yellowish-white halo with distinct edges and a central black dot; (d) stage IV: involution phase, characterized by a dark-brownish discoloration or black crust with a deceased parasite, with or without necrosis in the surrounding area; and (e) stage V: residual circular scarring [22]. Animals with active parasites in stages I, II, and III were considered positive. Animals with only stage IV and V inactive lesions were considered negative. The animals were also evaluated for acute clinical signs related to tungiasis lesions, such as hyperemia, pain, suppuration, clustering (three or more nearby lesions), fissure, ulcer, mutilation, lameness, and ectopic lesions.

### 2.3. Statistical Analysis

The factors associated with infection by *T. penetrans* were evaluated using a multivariate analysis. The cats were categorized by age range, sex, and coat length. These independent variables were compared with the dependent variable denoting the presence or absence of tungiasis, where stages I to III were considered positive (active lesions) and stages IV and V negative (inactive lesions), according to the Fortaleza classification. The respective data were submitted to multivariate regression analysis using the generalized multivariate model. For the selection of the independent variables, backward approximation was used. The best model was defined as having the lowest value of the Akaike information criterion, and variables were considered significant with a *p*-value < 0.05. For variables not included in the best model, a univariate analysis was performed. All statistical analyses were carried out with R software (version 3.6.1) using the *stats* (GLM function) and *epiDisplay* (logistic.display function) packages.

Cat coat length was determined with visual inspection based on cats of the defined breeds British Shorthair for short hair and British Longhair for long hair, according to the standard of the Fédération Internacionale Féline (FIFe) [39]. Age was categorized based on existing guidelines for standardizing the age range of felines: kitten, animals up to one year; junior, from one year to two years; prime, from three to six years; and mature, seven years or older [40,41]. The number of lesions of stages I to III by age range was also compared. For this, the normality test of the number of lesions was performed. Since the resulting distribution was not normal, the nonparametric Kruskal–Wallis test followed by the Wilcoxon post hoc test with Bonferroni correction for the *p*-value was performed. The result was considered significant with a *p*-value < 0.05. All analyses were performed with the R program (version 3.5) using the epiDisplay and rstatix packages. For chickens, a descriptive analysis was used.

## 3. Results

### 3.1. House Visits and Geolocation

A total of 272 houses were counted and visited. Of these, 41.6% (113/272) were closed because they were used only for tourism at certain times of the year, known locally as “summer houses”, so there were no animals.

Of the houses that had permanent residents, 29.5% (47/159) had no animals, while 30.1% (48/159) only had dogs, and 40.6% (64/159) had cats and chickens cohabiting with dogs. In 9 of the 64 residences, the cats could not be evaluated since they had feral behavior, with excessive aggression, making rendering and inspection impossible (not even the owners could approach them).

**Figure 2:**
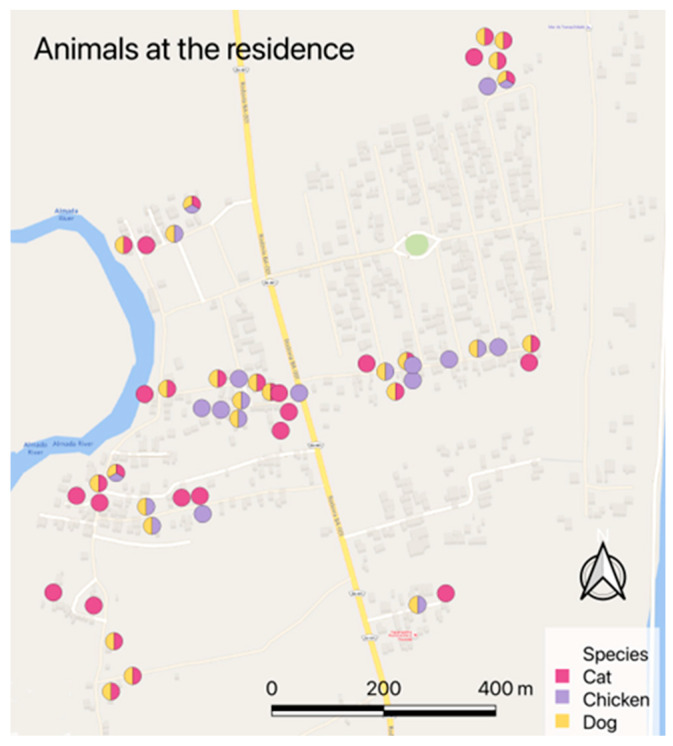
Residences with evaluated cats, dogs, and chickens.

Regarding spatial distribution, the residences with chickens were more frequently located in the central region of the community, but residences with cats were homogeneously distributed in space (Figure 2).

**Figure 3:**
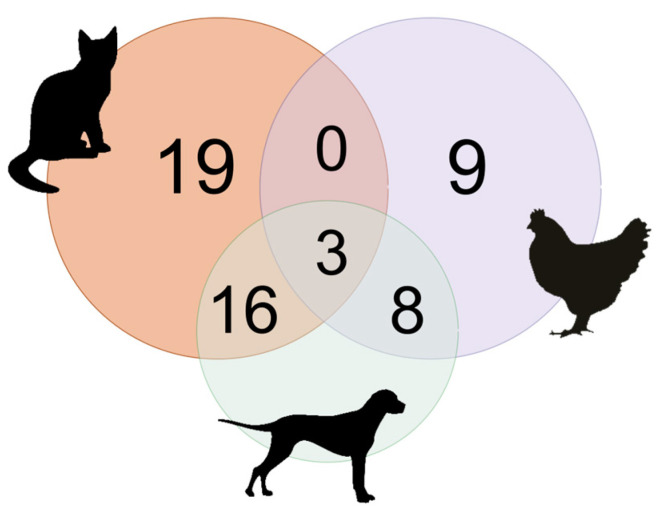
Venn diagram showing the number of houses that had different combinations of cats, dogs, and chickens.

Of the remaining 55 houses that had domestic animals, 34.5% (19/55) had only cats, while in 29.1% (16/55) of these houses, cats and dogs lived together. In 16.3% (9/55) of these residences had only chickens, while 14.5% (8/55) of these residences, chickens cohabited only with dogs. Only 5.5% (3/55) of these residences had dogs, cats, and chickens lived together (Figure 3).

In 7.3% (4/55) of these houses, other types of animals (six ducks, six tortoises, and one parakeet) were found living with cats, dogs, and/or chickens. There were no other animal species in the residences or in the neighborhood.

### 3.2. Evaluation of Cats in Vila Juerana

In total, 111 cats were counted, and 63.9% (71/111) were inspected. All cats were semi-domiciled and had free access to the street. Of these, 49.8% (35/71) were male and 50.2% (36/71) were female. Due to their feral behavior, as described above, the other 36.1% (40/111) of the cats could not be evaluated.

Of the cats evaluated, 38% (27/71; 95% 26.74–49.32) were positive for tungiasis lesions (stages I, II, and III), while 12.7% (9/71) had lesion stages IV or V and were considered negative, and 49.3% (35/71) had no lesions suggestive of tungiasis. The number of lesions per cat and the total number of lesions are listed in Table 1. The evaluation of neosomes using stereo microscopy revealed that the species affecting cats in Vila Juerana was *T. penetrans* [34].

**Figure 4:**
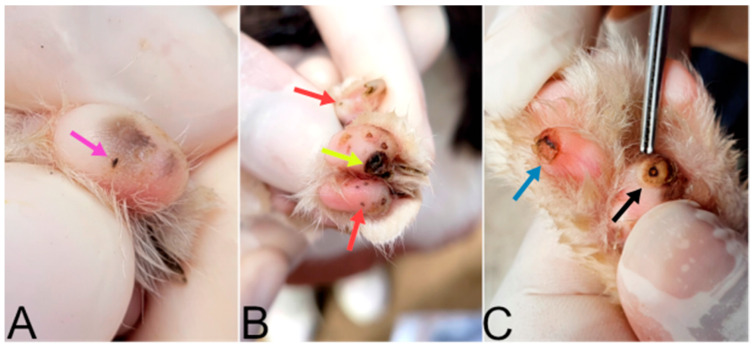
(**A**) Cat paws with a stage I lesion (pink arrow), which is the exact moment when the *Tunga penetrans* female penetrates its host. (**B**) Cat paw with stage II lesions (red arrows) and a stage V lesion (yellow arrow). (**C**) Cat paw with a stage III lesion (black arrow) and stage IV (blue arrow) lesion according to the Fortaleza Classification [22].

**Figure 5:**
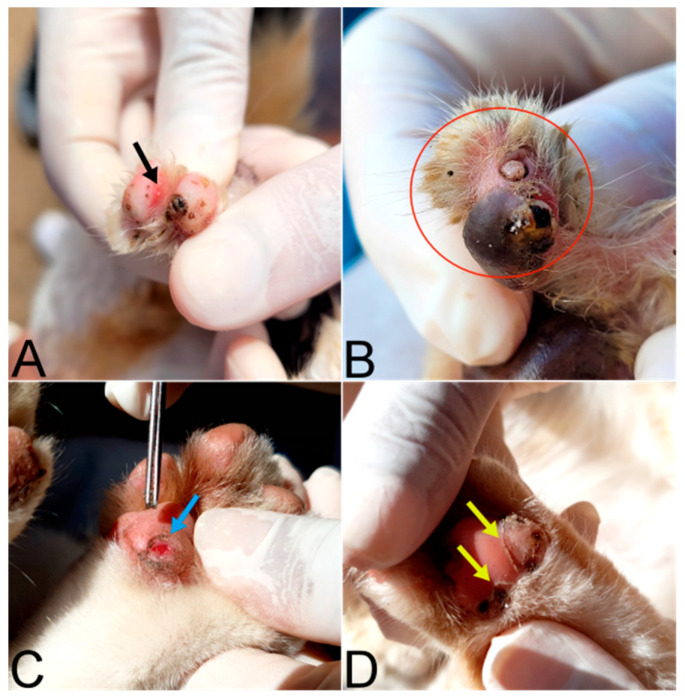
Clinical signs related to *Tunga penetrans* infection in cat paws from Vila Juerana. (**A**) Hyperemia (black arrow); (**B**,**C**) ulcerations (red circle and blue arrow, respectively); and (**D**) fissures (yellow arrows).

Lesions (Table 1 and Figure 4) resulting from all stages of development of *T. penetrans* were found, totaling 192 lesions. Mutilation was the most observed clinical sign in cats (Table 1 and Figure 5). All lesions in cats were in the periungual region and pads. No ectopic lesions were observed. A total of 77.7% (21/27) cats had more than one stage of injury, while 59.3% (16/27) showed no clinical signs, 40.7% (11/27) had clinical signs, and six had more than one associated clinical sign.

**Figure 6:**
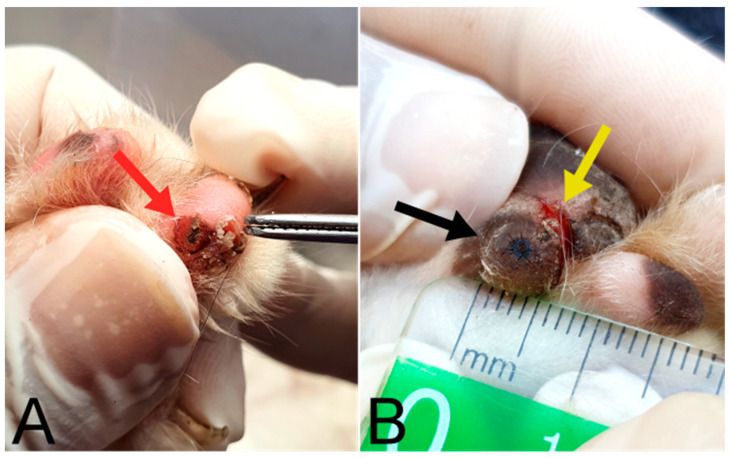
(**A**) Cat with stage III lesions on paws (red arrow) and mutilation (tweezers). (**B**) Stage III lesion approximately 0.5 cm in diameter (black arrow), and the presence of a fissure resulting from the lesion (yellow arrow) [22].

Regarding the acute and chronic clinical signs related to *T. penetrans* infection, signs of hyperemia, clustering, fissures, ulceration, and mutilation were found (Figure 5 and Figure 6). Other clinical signs such as lameness and suppuration were not observed in the animals.

Among the three variables related to the characteristics of the cats, only age and sex were included in the multivariate analysis, and coat length was included in the univariate analysis. There was only one animal in the mature category, so it was removed from all regression analyses. In these analyses (Table 2), 51.4% (18/35) of males and only 25.7% (9/36) of females had tungiasis, meaning that males were 3.75 times more likely to become infected than females, with a significant difference (*p* = 0.016; Table 2). Kittens and prime cats had a lower infection prevalence (22.1% and 26.7%, respectively) than juniors (47.6%), but there was no significant difference between these groups (*p* = 0.055). Similarly, long-haired animals had a higher infection prevalence (42.9%; 3/7) than short-haired animals but without statistical significance (*p* = 0.806).

### 3.3. Evaluation of Chickens in Vila Juerana

**Figure 7:**
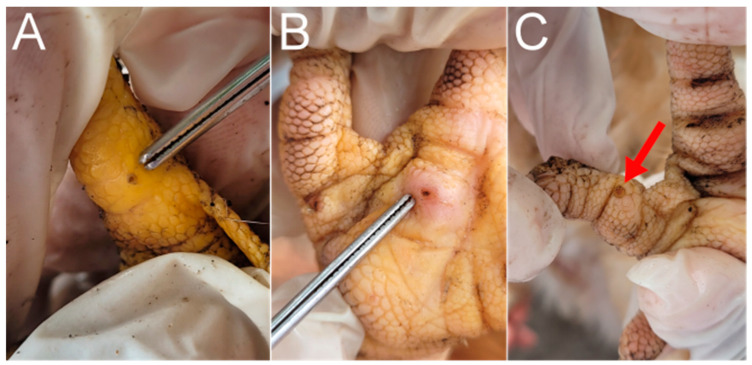
Feet of chickens infected with *T. penetrans*. (**A**,**B**) Stage II lesions (tweezers). (**C**) Stage IV lesion (red arrow).

The houses in Vila Juerana had a total of 439 chickens, of which 173 were inspected. Among these 173 chickens, 2.31% (4/173; 95% IC 0.07–4.5) had lesions caused by *Tunga* spp. (Figure 7).

**Figure 8:**
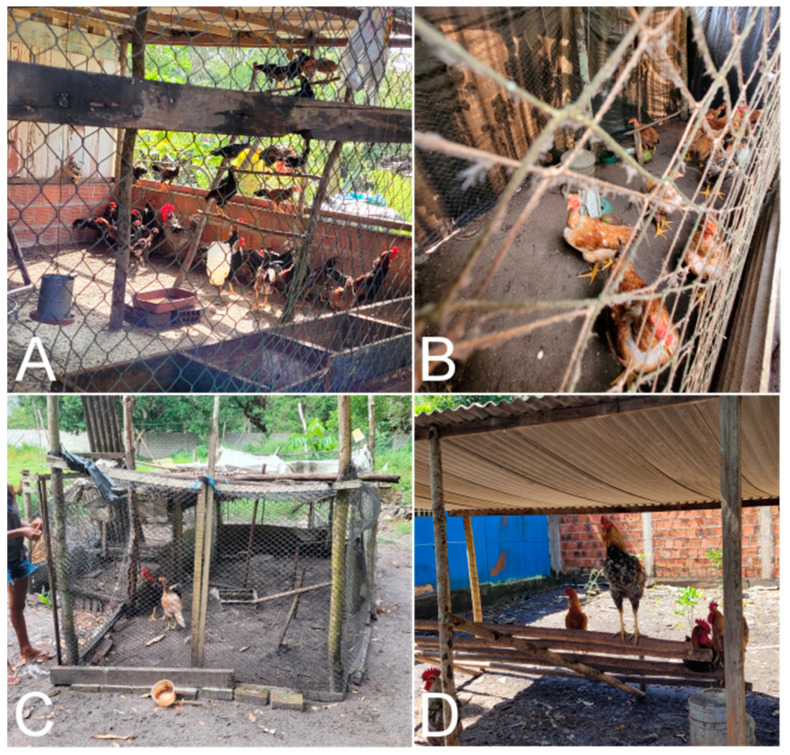
Chicken coops in Vila Juerana, Ilheus Bahia, Brazil. In one chicken coop, the floor was made of cement (**A**), while the other chicken coops had sandy floors (**B**–**D**).

None of the chicken coops had bedding. An investigation of the type of floor in the coops indicated the predominance of sandy floors, except in one house, where the chicken coop floor was made of cement (Figure 8). All chickens with *T. penetrans* were kept in coops with sandy floors.

### 3.4. The Evaluation of Other Animals

Six ducks, six tortoises, and one parakeet were also inspected. In the residences where ducks were found, they coexisted with dogs and chickens. The tortoises coexisted with cats, dogs, and chickens, and the parakeet lived with dogs and chickens. None of them were infected.

### 3.5. Assessment of Cohabitation in the Dwellings with Infected Cats and Chickens

**Figure 9:**
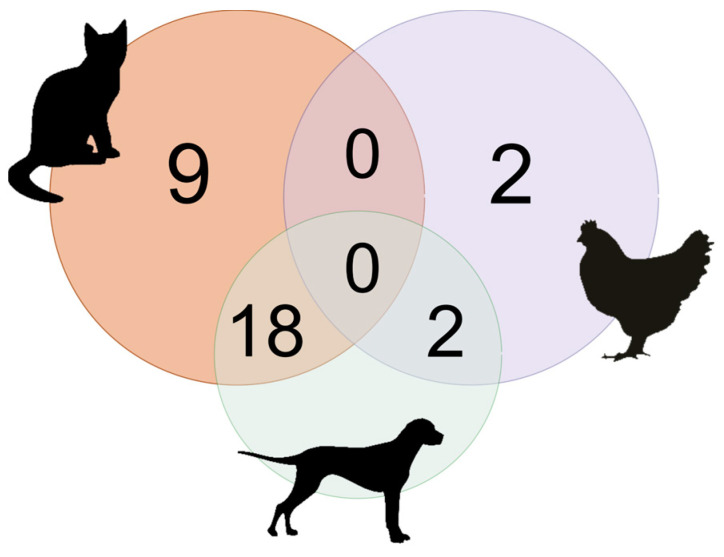
Venn diagram showing the number of positive cats and chickens that cohabited with positive dogs.

Of the 27 positive cats, 66.6% (18/27) cohabited with positive dogs and 33.3% (8/27) did not cohabit with any other positive animal. Of the four positive chickens, 50% (2/4) cohabited with positive dogs and the rest did not cohabit with any other species (Figure 9).

## 4. Discussion

Several studies have identified the participation of domestic cats in the epidemiological chain of tungiasis in Brazil [16,32,33,42,43,44,45]. In Fortaleza, state of Ceara, also located in the northeast region of the country, high infection rates were found in cats from a shanty town (slum) (49.6%) and from a fishing community (32.4%) [32]. This high prevalence was expected in our study (36.62%) due to similarity in the environmental, structural, and sanitary characteristics of the communities investigated. We also emphasize that in the studies cited above, none of the authors investigated stages of infection and clinical signs in domestic cats, which is essential for understanding the role of these animals in maintaining the endemic profile in communities since they act as egg dispersers.

In this respect, the previous reports of feline tungiasis [23,33] combined with the high prevalence and high number of active lesions found in this study indicate that domestic cats are important reservoirs of the parasite. Mutilation was the most commonly observed clinical sign in the cats from Vila Juerana, which was also observed in the community’s dogs [23]. This finding can be explained by feline hygiene and grooming habits to relieve itching and remove the parasite with repeated licking/biting [46], which can lead to the loss of nails or phalanges.

The prevalence of infection was higher in male cats. The natural behavior of semi-domesticated animals such as hunting [47], associated with searching for females in estrus and territory marking [47,48], leads male cats to travel greater distances and explore a wider variety of habitats, creating a higher probability of exposure to fleas. In turn, this increases the risk of infection and spreading flea eggs inside and outside residences [49].

A relevant factor for the high prevalence of tungiasis in felines in this study is the fact there are many feral cats in the village, which are not accessible for evaluation and treatment. The lack of adequate treatment for tungiasis in cats associated with the large feral population makes these animals important vectors of tungiasis, equal to or even greater than dogs. Even with tungiasis control in dogs, this cat population contributes to the spread of *T. penetrans* in the environment [50]. We suppose that this same factor may be associated with the lack of seasonality in the disease in the region, considering there is no treatment for cats and that dogs are rarely treated in the community. Thus, the absence of tungiasis control in cats and cohabitation with dogs in residences may be a limiting factor for canine prevention of human tungiasis [6,23,33,38,51].

Chickens have a less relevant role in tungiasis as incidental hosts in Vila Juerana. However, inspection of these animals is recommended since they live in the same environment as pigs, dogs, cats, and humans in endemic areas [44,50]. In a study that inspected chickens in Rio de Janeiro, Brazil, none were found to be infected with tungiasis [30]. Studies in Kenya have associated *Tunga* spp. infection in humans with the presence of chickens and other domestic animals, mainly dogs [26,52]. In the present study, one of the houses with positive chickens also had positive dogs. There were no dogs in the other residence with infected chickens. However, the sandy soil in the chicken coops and the habit of raising semi-domesticated animals in the village, allows for an interaction between positive dogs and chickens, increasing the risk of transmission and dissemination of *T. penetrans* between species [53]. Based on our field observation, we believe this low incidence of tungiasis in chickens can be explained by the fact that chickens normally scratch the sandy ground looking for food (foraging behavior) [53], and consequently, may ingest some reproductive forms of parasites directly from the soil. Another important factor is that their feet are made of thick keratin plates [54], which makes it difficult for the flea to penetrate. In some feet, lesions like mutilations by pecking were found, and we believe this possibly indicates the elimination of the neosomes by the animals’ beaks.

Although dogs are considered the main reservoirs of *Tunga* spp. in Brazil [23], there is a need for further investigation regarding felines and other animal species and their potential epidemiological risks [16,30,32,42] since these species have constant interactions. Additionally, since semi-domesticated or feral cats can cover large geographical areas and promote the dissemination of eggs, it is important to understand how cats contribute to the renewal of the life cycle and maintenance of this zoonosis in endemic areas. Furthermore, the spread of *T. penetrans* by reservoir animals like cats may be a risk factor for accidental infections of tourists who carry the disease back to their home regions [19,55].

Urgent government actions aimed at educating cat owners regarding management guidelines to control the population of feral cats are necessary for the control of diseases such as tungiasis. Rigorous sanitary control of animals living in the same environment is also necessary to combat the spread of fleas between species.

## 5. Conclusions

In the region of Vila Juerana, Bahia, Brazil, cats have a high incidence of tungiasis, constituting the second most important host of *T. penetrans* in the region. With regard to chickens, although they are part of the same environment where infected dogs and cats circulate and spend most of their time on sandy soil, the results of this study suggest they play a less important role in the dissemination of *T. penetrans* and are thus accidental hosts in the region studied.

## Figures and Tables

**Table 1:** Staging (Fortaleza Classification) [22] and quantification of lesions associated with clinical signs in infected cats (n = 27) from Vila Juerana, Ilhéus, Bahia, Brazil.

	Category	Infected Animals (n)	Total of Lesions
Lesion stages	I	2	2
	II	20	62
	III	22	56
	IV	14	31
	V	17	22
Total		27 *	173
Clinical signs **	Mutilation	6	7
	Cluster	5	5
	Hyperemia	4	4
	Ulceration	2	2
	Fissure	2	2
Total number of infected		27	20

Overall * 21 cats presented more than one lesion stage; ** 16 cats had no clinical signs; 11 cats had clinical signs; and 6 had more than one associated clinical sign.

**Table 2:** Number and percentage of positive and negative cats for each variable and their respective results of the univariate and multivariate regression analyses.

		Positive	n%	Negative	n%	Total	OR 95% IC	*p*-Value	Analyses
Age	Kitten	3	23.1	10	70.9	13	0.56 (0.08, 3.39) Ref 0.41 (0.09, 1.49)	0.055 0.192	Multivariate
	Junior	20	47.6	22	52.4	42			
	Prime	4	26.7	11	73.3	15			
Sex	Female	9	25.7	26	74.3	35	Ref 3.75 (1.32, 11.51)	0.016 *	Multivariate
	Male	18	51.4	17	48.6	35			
Coat length	Short	24	37.15	39	62.9	63	Ref 1.22 (0.22, 5.99)	0.806	Univariate
	Long	3	42.9	4	57.1	7			

* Significant *p* (*p* < 0.05); OR: odds ratio; n: number of animals in the category.

## Data Availability

All data are available within the article.
